# Congenital bronchopulmonary malformations: A single-center experience and a review of literature

**DOI:** 10.4103/1817-1737.43080

**Published:** 2008

**Authors:** Basant Kumar, Leela D. Agrawal, Shyam B. Sharma

**Affiliations:** *Department of Pediatric Surgery, Sir Padampat Mother and Child Health Institute, (JayKayLon Hospital), S.M.S. Medical College, Jaipur-302 004, Rajasthan, India*

**Keywords:** BC, CCAM, CLE, congenital bronchopulmonary malformations, lobectomy, PS

## Abstract

**PURPOSE:**

To present a single-center experience with 25 cases of bronchopulmonary malformations and the review the literature.

**MATERIALS AND METHODS:**

We conducted a retrospective analysis of the medical records of patients with congenital bronchopulmonary malformations who were operated between July 1997 and July 2007 in our institute; we examined the modes of presentations, management, and outcome. Outcome of all patients was assessed over a short follow-up period (average 1.8 months).

**RESULTS:**

Out of 25 patients, 18 (72%) were male and 7 (28%) were female. Age of patients ranged from 1 day to 11 years. The histopathological diagnosis was congenital cystic adenomatoid malformations [CCAM; n = 14 (56%)], congenital lobar emphysema [CLE; n = 5 (20%)], pulmonary sequestrations [PS; n = 3 (12%)], and bronchogenic cysts [BC; n = 3 (12%)]. Antenatal diagnosis was available in only 2 (8%) patients. The common presenting symptoms were respiratory distress and chest infections. Lobectomy was the procedure of choice . Mortality was 16% (n = 4; M: F = 3: 1). Two patients died because of overwhelming sepsis, one from compromised cardiac function, and one from aspiration which might possibly have been prevented.

**CONCLUSION:**

Patients with progressive respiratory distress due to these anomalies may require urgent surgical intervention regardless of age. The surgical outcome is favorable, with manageable complications. Plain x-ray chest and CT of thorax are usually sufficient for diagnosis and planning of treatment. Pathological diagnosis may differ from the imaging diagnosis. Mortality is found to be more in neonates. Apart from initial stabilization, resection of lesion and careful postoperative care is necessary to reduce mortality and morbidity.

Congenital malformations of the lung and mediastinum are relatively rare and vary widely in their presentation and severity. Most of these lesions are cystic in nature, but some are solid and others may have both components.[1] These developmental abnormalities of the foregut, tracheobronchial tree, and pulmonary parenchyma constitute a variety of benign lesions in newborns, infants, and children. These bronchopulmonary anomalies are usually detected in the neonatal period or in early childhood. However, some are not encountered until later in childhood or in adulthood and some of them may even remain asymptomatic throughout life.[2] Some of these anomalies can be confused with more sinister abnormalities and may be incompatible with life; requiring immediate intervention. The clinical presentation in children can range from acute respiratory distress to refractory chronic infective disease. With advances in antenatal diagnosis, management of such cases can begin while the fetus is still in the mother's womb.[2–4] In developing countries, a large number of children with congenital benign lesions of lung and mediastinum are inially seen and treated in peripheral hospitals before being referred to tertiary level pediatric centers and so they may present very late, hence the delay in diagnosis and in initiation of proper treatment.

This retrospective study was undertaken to present our experience of 25 cases of congenital bronchopulmonary anomalies, their management, and the outcome in symptomatic patients after surgical intervention in our institute, a tertiary level pediatric center. We also present a review of the literature.

## Materials and Methods

All symptomatic patients with congenital bronchopulmonary anomalies, operated during July 1997 to July 2007 at our institute [Sir Padampat Mother and Child Health Institute (JayKayLon Hospital), S.M.S. Medical College, Jaipur, Rajasthan, India], were included in the study. Patients who were not operated and died before surgery were excluded. The study is based on the data available in hospital records. We collected information related to clinical presentation, diagnostic parameters, operative findings, histopathology of resected lesion, postoperative course, and final outcome. The follow-up period ranged from 1–5 months (mean: 1.8 months).

The commonest indication for surgery was chest infection and respiratory distress. Two patients with congenital lobar emphysema and one patient with congenital cystic adenomatoid malformation had intercostal tubes inadvertently inserted for suspected pneumothorax before surgical referral. Imaging with x-ray chest and abdomen alone or with an additional computed tomography (CT) scan was performed in all patients. Water-soluble contrast (gastrograffin) upper GI study was done in three patients with congenital cystic adenomatoid malformation to exclude congenital diaphragmatic hernia. In these patients, CT scan was not done. Preoperative ventilatory support was required in four (16%) patients with severe respiratory distress and cyanosis. All these patients were below 1 month of age. Antenatal diagnosis was available in only 2 (8%) patients with congenital cystic adenomatoid malformations (CCAM).

## Results

From July 1997 to July 2007, 25 patients with congenital bronchopulmonary malformations were operated in our institute. In this series, males predominate with a male : female ratio of 2.6: 1 [male: 18 (72%) and female: 7 (28%)]. The age of patients ranged from 1 day to 11 years. Twenty patients (80%) were less than 1 year old, while 12 patients (48%) were below 1 month of age. Out of 25 patients, 23 patients (92%) had pulmonary and two patients (8%) had mediastinal lesions. The details of the type of lesions, sex distribution, presenting symptoms, age at surgery, procedure performed, and outcome are shown in Table 1. Out of 25 patients operated for bronchopulmonary developmental anomalies, four patients died in the postoperative period (overall mortality rate: 16%). All patients who died (all with CCAM) were below 1 month of age. Of the four patients who died, three were boys and one was a girl.

**Table 1:** Congenital lesions, number, sex distribution, age of patients at surgery, presentation, site of lesion, surgery, and outcome (*n* = 25)

Pathology	No. of cases (%)	Age at surgery	Sex M : F	Presenting symptoms	Site of lesion# (No. of patients)	Surgery	Outcome
Congenital cystic adenomatoid malformation	14 (56)	2–75 days (mean age: 11.6 days)	11 : 3	Cyanosis, respiratory distress, chest infection	LLL; (6)	Lobectomy of affected lobel(s)	4 patients died (28.6%) Others recovered
					LUL; (2)		
					RLL; (3)		
					RUL; (1)		
					RML + RLL; (2)		
Congential lobar emphysema	5 (20)	1.5 months;	3 : 2	Dyspnea, cyanosis, wheeze, respiratory distress, chest infection	LUL; (3)	Lobectomy of affected lobel(s)	All recovered
		2 months;			RU; (1)		
		3.5 months;			RML; (1)		
		3 Years;					
		8 Years;					
Pulmonary sequestration	3 (12)	20 days;	1 : 2	Respiratory distress, fever, cough	LLL; (2)	Lobectomy of affected lobel(s)	All recovered
		1 month;			RUL; (1)		
		2 months.					
Bronchogenic cyst	3 (12)	1 year;	3 : 0	Cought, wheeze, chest infection;	Mediastinal	Excision of cyst;	All recovered
		4 Years;			(rt. side); (2)	RL lobectomy	
		11 Years		fever	Pulmonary (RLL); (1)		

^#^*LLL:* left lower lobe, *LUL:* left upper lobe, *RLL:* right lower lobe, *RML:* right middle lobe, *RUL:* right upper lobe, Overall mortality was 16% (*n* = 25).

Histopathology of the resected lung tissue in 14 (56%) patients showed CCAM [Table 2]. The plain x-ray chest or / and CT thorax had shown signs of cystic lung disease and localized the affected lobe(s). One patient had an intercostal tube inserted before surgical referral. Three patients (12%) required preoperative ventilatory support and five patients (20%) needed it postoperatively; all of them were below 1 month of age. One patient was misdiagnosed as congenital diaphragmatic hernia (CDH) and underwent a negative laparotomy; subsequently, thoracotomy and lobectomy was performed. CT scan was not done in this case. Latter, contrast upper GI study was performed in three other patients to exclude CDH. In one patient, congenital lobar emphysema (CLE) was suspected preoperatively but histopathology revealed CCAM. Four patients died in the postoperative period; all were below 1 month of age and required ventilatory support. All other children recovered, with the average duration of hospital stay being 13 days (range: 8–21 days).

**Table 2:** Discrepancy between preoperative (radiological) and histological diagnosis

Pathology	Radiological investigations (No. of patients)	Preoperative diagnosis (No. of patients)	Histological diagnosis	Remark
Congential cystic adenomatoid malformations (CCAM)(*n* = 14)	X-ray + CT scan: (8)	CCAM in all	CCAM in all	Preoperatively, two patients with CCAM were misdiagnosed as CLE and diaphragmatic hemia on the basis of chest x-rays only
	X-ray only: (3)	CCAM: (1)	CCAM in all	
	X-ray + upper Gl	CLE: (1)	CCAM in all	
	contrast study: (3)	Diaphragmatic hernia; (1)		
		CCAM in all		
Congenital lobar emphysema (CLE) (*n* = 5)	X-ray + CT scan: (3)	CLE in all	CLE in all CLE in both	—
	X-ray only: (2)	CLE in both	CLE in both	
Pulmonary sequestrations (PS) (*n* = 3)	X-ray + CT scan in all	IPS*: (1)	IPS	Two patients with PS were misdiagnosed as CCAM preoperatively, in spite of both x-ray and CT scan
		CCAM: (2)	IPS in both	
Bronchogenic cysts (BC) (*n* = 3)	X-ray chest + CT scan in all	BC: (2)	BC in both	One patient with BC was misdiagnosed as CCAM preoperatively, in spite of CT scan
		CCAM: (1)	BC	

* IPS: Intrapulmonary sequestrations.

There were five (20%) histologically proved cases of CLE. Two patients had intercostal tube in situ before surgical referral. X-ray chest and CT scan showed a hyperinflated lung lobe with mediastinal shift to the opposite side and atelectasis of the neighboring lobes. Lobectomy of the affected lobe was performed in all. One patient required postoperative ventilatory support. All patients recovered, with satisfactory expansion of the remaining normal lung tissue in follow-up chest X-rays. The average duration of hospital stay was 12 days.

There were three (12%) cases of intralobar pulmonary sequestration (IPS) that presented with respiratory distress, cough, and fever. Chest X-ray and CT scan showed a cystic lesion involving the left lower lobe in two patients and the right upper lobe in one patient. Diagnosis of IPS in one patient and CCAM in two patients was made on the basis of X-ray and CT scan findings. Lobectomy of affected lobe(s) was performed and histopathology confirmed the diagnosis of IPS instead of CCAM. All patients recovered well and were discharged on the tenth postoperative day.

Histopathologically, three (12%) patients were diagnosed to have bronchogenic cyst and presented with complaints of recurrent chest infections, cough, fever, and wheeze. Two patients (4 years and 11 years old) had mediastinal cysts situated in the right side of the chest and one patient (1 year old) had an intrapulmonary bronchogenic cyst involving the right lower lobe. It was diagnosed as CCAM preoperatively on the basis of the CT scan but histology showed it to be a intrapulmonary bronchogenic cyst. Thoracotomy and excision of the mediastinal cyst and right lower lobectomy for the intrapulmonary cyst was done. All patients recovered, but one child (the 4-year-old) with an infected cyst developed a bronchopleural fistula and the subsequent chronic empyema required decortications 3 months after surgery. The average duration of hospital stay was 15 days.

Postoperative complications encountered were wound infection (3), pneumonitis (6), atelectasis (1), hydropneumothorax / pneumothorax (3), air leak (1), and empyema (1). These were all managed successfully by conservative means, except for the empyema which needed decortications. Overall, six patient required postoperative ventilatory support, the duration ranging from 1–5 days (average 2 days). There were four postoperative deaths found in this series: all in CCAM. Two patients died due to overwhelming sepsis and one child died because of aspiration on the fifth day; this might have been prevented. One patient with CCAM died within 2 h of surgery due to an associated cardiac anomaly that was only diagnosed during surgery. The rest of the patients recovered uneventfully and were doing well over the short follow-up period.

## Discussion

Congenital malformations of the lung and mediastinum are relatively rare.[1] These lesions often present in the newborn period with respiratory distress, and require prompt diagnosis with emergency surgical resection.[2] The etiology of such lesions is controversial and many theories have been proposed.[5] The cause of many of these malformations has been described as in-sequestration and foregut malformations, whereas that of others (e.g., CCAM and simple lung cyst) remain obscure.[1] During fetal lung growth, the timing of embryologic alterations, whether early or late, determines not only the type of lesion but also the severity of the malformations and its impact on overall lung growth.[6]

CCAM was diagnosed in 14 of the 25 patients (56%) and represent the most common congenital bronchopulmonary foregut malformation in our series. It probably results from a cessation of bronchomaturation and concomitant overgrowth of mesenchymal elements at about the fifth to sixth week of gestation.[7] About 15–50% of CCAMs decrease in size significantly before birth.[8] Stocker *et al.*[9] classified CCAMs into three types on the basis of their pathological characteristics, primarily the cyst size. Adzick *et al.*[10] classified them into macrocystic type (cyst size 5 cm or more) and microcystic type (cyst size < 5 cm) on the basis of gross anatomy and ultrasound findings. In our series, nine patients had macrocystic and five had microcystic type pathology. The majority of CCAM lesions can be diagnosed antenatally,[2,4] but in our series only two (8%) patients had been diagnosed antenatally with ultrasound. Chest x-ray may appear normal in early neonatal life[2,3] and this does not exclude the presence or regression of CCAM.[7] CT scan is mandatory if the size and extent of lesion has to be evaluated with 100% sensitivity.[7] We also recommend postnatal CT imaging in all children with suspected CCAM. It usually involves a single lobe of a lung and bilateral cases are rare.[2,10] In our series, there was no bilateral case. Most of the symptomatic patients present within the first year of life[2,7] and symptoms vary from respiratory distress to chronic cough and recurrent chest infection. All our patients were symptomatic and presented within 3 months of birth. All patients had unilateral disease involving a single lobe, except for one patient who had involvement of two lobes (RML and RLL). The most common presenting symptom was respiratory distress and cyanosis. Lobectomy of affected lobe(s) was performed in all patients and is the surgical procedure of choice for most lesions.[1] Stover *et al.*[11] recommended surgical resection in asymptomatic patients with CCAM in the first year of life because of the potential risk of complications. The main reason for surgical treatment in an asymptomatic patient is the potential for complications such as recurrent infection in form of pneumonia or lung abscess, pneumothorax, and malignant transformation.[4,12] Rhabdomyosarcoma and bronchoalveolar carcinoma have been noted as complications of CCAM.[4,12] Prenatal diagnosis is useful for the planning of postnatal treatment. There was satisfactory outcome following surgery (including neonatal emergency surgery), with a mortality of 4 out of 14 patients with CCAM (mortality rate: 28.57% in CCAM). This may seem to be on the high side but it must be remembered that one patient died due to aspiration (which might have been prevented) and three other patients had very poor general condition on presentation and needed ventilatory support; in addition, one child had an associated cardiac anomaly.

CLE is characterized by hyperinflation of pulmonary lobe(s) due to air trapping with concomitant compression of surrounding structures. The exact cause of CLE is difficult to determine and no apparent cause is found in 50% of cases.[13] The most commonly identified cause is a congenital defect of cartilage, ranging from hypoplastic and flaccid tissue to complete absence, which accounts for 25% of cases.[13] The remaining 25% have other causes of bronchial obstruction, such as redundant mucosal fold, mucus plugging, anomalous cardiopulmonary architectures and, rarely, intrathoracic masses.[13] CLE is more common among boys than girls and is usually unilateral, commonly affecting the LUL followed by the RML and the RUL; bilateral cases are also reported.[13] The earlier the age of presentation, the more severe is the respiratory distress and the greater is the need for surgery.[2,13] Contrary to previous belief, CLE is not limited to Caucasians, and it seems to affect other races as well.[2,14] The presence of diminished lung markings within a radiolucent area on a plain x-ray of chest, with evidence of collapse of the adjacent lobe, differentiates CLE from pneumothorax.[2] CT scan of the chest may show intrinsic or extrinsic bronchial obstruction.[2,13] In our series of five patients, two patients had had intercostal tube insertion done for suspected pneumothorax before surgical referral; however, none of them actually had a pneumothorax. We recommend that intercostal tube insertion should be avoided because it further increases respiratory distress and causes pain during respiration. One child (1.5 month old) presented with severe respiratory distress, wheezing, and cyanosis. Emergency left upper lobectomy was performed. Bronchoscopy was done in this child before surgery because of suspicion of foreign body aspiration but no obvious foreign body or bronchial abnormality was found. Symptoms are generally more severe in upper lobe disease than in middle lobe disease.[2,14] In our series, three patients had disease in the left upper lobe (LUL), one in the right upper lobe, and one in the right middle lobe (RML). Lobectomy of affected lobe(s) was performed in all these cases and is the surgical treatment of choice for CLE.[2,14,15] One patient (1.5 months old) needed postoperative ventilatory support; a satisfactory outcome was observed in this case. Infants older than 2 months presenting with mild to moderate respiratory symptoms associated with normal bronchoscopic findings can be treated conservatively.[13,14] About 20% of patients with CLE have been reported to have associated congenital cardiac anomalies,[3] but in our series cardiac anomalies were absent.

Pulmonary sequestrations (PS) are masses of nonfunctioning lung tissues that are supplied by an anomalous systemic artery and do not have communication with the bronchoalveolar tree.[2] Sequestration has an estimated incidence of 0.15–1.7% in the general population.[8] Intrapulmonary sequestrations (IPS) are two or three times more frequent than extrapulmonary sequestrations (EPS), occur more frequently in males, and are mostly localized in the left posterior mediastinum.[2,8] Unlike IPS, EPS is usually asymptomatic and is found incidentally, but associated anomalies like diaphragmatic hernia or eventration, tracheoesophageal fistula, etc., may occur in 4% of cases.[2,6,8] In our series, all children had intrapulmonary sequestration (LLL and RUL) without any associated anomaly. Two of them were females. The lobectomy of the affected lobe(s) was performed in all cases with favorable outcomes. Two patients were diagnosed as CCAM preoperatively but histopathology confirmed the diagnosis of pulmonary sequestrations.

Bronchogenic cysts (BC) are congenital benign masses commonly located in the mediastinum or lung parenchyma; they arise from anomalous budding of the primitive tracheobronchial tube.[16] These cysts occur in the mediastinum or anywhere in the pulmonary parenchyma and may be present at subpleural, pericardial, paravertebral, and cervical locations.[6,16] These cysts tend to be asymptomatic in older children and adults. The common presenting symptoms are dyspnea, cyanosis, cough, chest infection and, rarely, dysphagia. In our series, there were two patients with mediastinal cysts who presented with nonproductive chronic cough and recurrent bouts of fever. One patient with a pulmonary cyst presented with dyspnea and history of recurrent chest infections and was diagnosed as CCAM preoperatively. Complete excision was performed in all cases with favorable outcomes. There were no unusual manifestations like bronchial atresia, superior vena cava syndrome, pericardial compression, cardiac arrhythmias, or hypoplasia of pulmonary artery in our series, though this has been described as occurring secondary to bronchogenic cysts.[16]

On the basis of data from these patients with congenital bronchopulmonary malformations, we can conclude that although these anomalies are comparatively rare, they pose significant morbidity and mortality in neonates and infants, especially in developing countries where resources are limited. Neonates, infants, and children presenting with respiratory distress due to these developmental anomalies may require urgent surgical intervention. The surgical outcome is favorable, with manageable complications. Plain x-ray chest and CT scan of the thorax are usually sufficient for diagnosis and planning of treatment but pathological diagnosis may differ from the imaging diagnosis. Accurate preoperative diagnosis can prevent inadvertent intercostal tube insertion. Lobectomy is the procedure of choice in our series. Our findings indicate that mortality is higher in neonates. Apart from initial stabilization, resection of lesion and careful postoperative care is necessary for improvement of the respiratory status in neonates and infants. Progressively increasing respiratory distress that shows no response to conservative treatment is an absolute indication for surgery, regardless of age; it would seem that emergency thoracotomy is tolerated in infancy and early childhood and even in neonates.
